# 25-hydroxycholesterol aggravates oxygen-glucose deprivation/reoxygenation-induced pyroptosis through promoting activation of NLRP3 inflammasome in H9C2 cardiomyocytes

**DOI:** 10.1590/1414-431X2024e13299

**Published:** 2024-05-03

**Authors:** Tao Jiang, Yong Li

**Affiliations:** 1 Department of Cardiovascular Medicine, Chongqing Hospital of Traditional Chinese Medicine, Chongqing, China

**Keywords:** CH25H, 25-hydroxycholesterol, NLRP3 inflammasome, Pyroptosis, H9C2 cardiomyocytes

## Abstract

25-hydroxycholesterol (25-HC) plays a role in the regulation of cell survival and immunity. However, the effect of 25-HC on myocardial ischemia/reperfusion (MI/R) injury remains unknown. Our present study aimed to investigate whether 25-HC aggravated MI/R injury through NLRP3 inflammasome-mediated pyroptosis. The overlapping differentially expressed genes (DEGs) in MI/R were identified from the GSE775, GSE45818, GSE58486, and GSE46395 datasets in Gene Expression Omnibus (GEO) database. Gene ontology (GO) and Kyoto Encyclopedia of Genes and Genomes (KEGG) pathway enrichment analyses were conducted using the database of Annotation, Visualization and Integration Discovery (DAVID). The protein-protein interaction (PPI) network of the overlapping DEGs was established using the Search Tool for the Retrieval of Interacting Genes (STRING) database. These bioinformatics analyses indicated that cholesterol 25-hydroxylase (*CH25H*) was one of the crucial genes in MI/R injury. The oxygen-glucose deprivation/reoxygenation (OGD/R) cell model was established to simulate MI/R injury. Western blot and RT-qPCR analysis demonstrated that CH25H was significantly upregulated in OGD/R-stimulated H9C2 cardiomyocytes. Moreover, knockdown of CH25H inhibited the OGD/R-induced pyroptosis and nod-like receptor protein 3 (NLRP3) inflammasome activation, as demonstrated by cell counting kit-8 (CCK8), lactate dehydrogenase (LDH), RT-qPCR, and western blotting assays. Conversely, 25-HC, which is synthesized by CH25H, promoted activation of NLRP3 inflammasome in OGD/R-stimulated H9C2 cardiomyocytes. In addition, the NLRP3 inhibitor BAY11-7082 attenuated 25-HC-induced H9C2 cell injury and pyroptosis under OGD/R condition. In conclusion, 25-HC could aggravate OGD/R-induced pyroptosis through promoting activation of NLRP3 inflammasome in H9C2 cells.

## Introduction

Myocardial reperfusion therapies such as percutaneous coronary intervention, thrombolysis, and other treatments are the major methods to restore coronary blood flow in patients with acute myocardial infarction (1). Timely and effective reperfusion can repair ischemic injury and reduce infarction size (2). However, the reperfusion process might aggravate myocardial damage and even cause cardiomyocyte death, a phenomenon known as myocardial ischemia/reperfusion (MI/R) injury (3). Accumulating evidence has shown that the MI/R injury is closely related to oxidative stress, calcium overload, inflammation and energy metabolism dysfunction (4), but the precise molecular mechanism underlying MI/R injury is still largely unknown.

Pyroptosis is a form of programmed cell death, which can be mediated by two signaling pathways (5). The classical pathway is characterized by the inflammasome activating cysteinyl aspartate-specific proteinase (caspase)-1, while the non-classical pathway is characterized by lipopolysaccharide activating caspase-4, caspase-5, and caspase-11. The Nod-like receptor protein 3 (NLRP3) inflammasomes can activate caspase-1, which facilitates the activation of gasdermin D (GSDMD), interleukin (IL)-1β, and pyroptosis (6). The NLRP3 and caspase-1 expression are increased in myocardial infarction, and knock-out of NLRP3 could reduce infarct size in different *in vivo* and *ex vivo* models of ischemia/reperfusion (7,8).

Oxysterol 25-hydroxycholesterol (25-HC), a synthetic product of cholesterol 25-hydroxylase (CH25H), plays multiple roles in lipid metabolism and immunity. Previous studies have shown that 25-HC can maintain mitochondrial integrity, suppress the absent in melanoma 2 (AIM2) inflammasome activation, and act as an important mediator on inflammasome activity and IL-1 family cytokine production (9,10). However, 25-HC exerts pro-inflammatory or anti-inflammatory effects based on different cellular and immunological conditions. Some studies demonstrated that 25-HC promoted inflammation through increasing release of cytokines, activation of NLRP3 inflammasomes, and inducing pyroptosis (11,12). Although various studies have yielded different results, the role of 25-HC in MI/R injury remains unclear. Therefore, this study aimed to investigate the role of 25-HC in MI/R injury and the potential mechanisms of this effect.

## Material and Methods

### Microarray datasets

The datasets GSE775, GSE45818, GSE58486, and GSE46395 were obtained from the Gene Expression Omnibus (GEO) database. C57BL/6 adult male mice (11-12 weeks old) were used in GSE775 and the 12-to-16-week-old male mice were used in GSE45818 (13,14). The MI/R experiments were performed in 12-13-week-old mice in GSE58486 (15) and adult male C57BL/6 mice were used in GSE46395 (16). A total of 32 samples were available, including 16 normal heart samples and 16 MI/R heart samples. The MI/R heart samples of four datasets were acquired from mice with left anterior descending coronary artery occlusion and reperfusion. The mice with MI/R injury had a larger infarct size on the left ventricle (LV). The raw data were normalized, and then the gene expressions in normal and MI/R heart samples were compared using GEOquery package.

### Identification of differentially expressed genes and gene ontology enrichment analysis

The fold change (FC) of gene expression between MI/R and normal samples was obtained, and the false discovery rate (FDR) was calculated through the Benjamini and Hochberg procedure using GEO2R tools. The differentially expressed genes (DEGs) were identified with a | log FC | >1 and an FDR <0.05. Furthermore, overlapping DEGs were determined by analyzing the DEGs from GSE775, GSE45818, GSE58486, and GSE46395. Gene ontology (GO) and Kyoto Encyclopedia of Genes and Genomes (KEGG) pathway enrichment analyses were conducted using the Database of Annotation, Visualization and Integration Discovery (DAVID; version 6.8; https://david.ncifcrf.gov/). The cutoff of significantly enriched GO terms and KEGG pathways was FDR <0.05.

### Protein-protein interaction network construction

The protein-protein interaction (PPI) network of overlapping DEGs was constructed using the Search Tool for the Retrieval of Interacting Genes (STRING; version 11.0; http://string-db.org/) database. The protein interaction pairs with a combination score >0.4 were analyzed for the construction of the PPI network. The integrated interaction information was visualized and downloaded from STRING.

### Cell culture

H9C2 cardiomyocytes were purchased from the National Collection of Authenticated Cell Cultures (China). The complete cell culture medium was prepared by adding 100 U/mL penicillin and streptomycin and 10% (v/v) fetal bovine serum (FBS) to high glucose Dulbecco's modified Eagle's medium (DMEM) (Hyclone, USA) (4.5 g/L of glucose). The culture medium was replaced every two to three days. Cells were cultured in a humidified 5% CO_2_ incubator (ESCO, Singapore) at 37°C. The cells from passages 10 to 15 were used for the next experiments.

### Induction of oxygen-glucose deprivation/reoxygenation

H9C2 cardiomyocytes were subjected to 6 h of oxygen-glucose deprivation (OGD) and 12 h of reoxygenation. Cardiomyocytes were first cultured in serum-free high glucose DMEM overnight before oxygen-glucose deprivation/reoxygenation (OGD/R). Then, the cells of OGD/R groups were incubated in DMEM without FBS and glucose for 6 h in a hypoxic chamber (Mitsubishi, Japan) at 37°C. Finally, the cells were reoxygenated for 12 h by incubation in high glucose DMEM with 1% FBS under normoxic conditions. Cardiomyocytes of the control group were incubated in high glucose-DMEM with 1% FBS under normoxic conditions for 18 h.

### Treatment of cells

**Figure 1:**
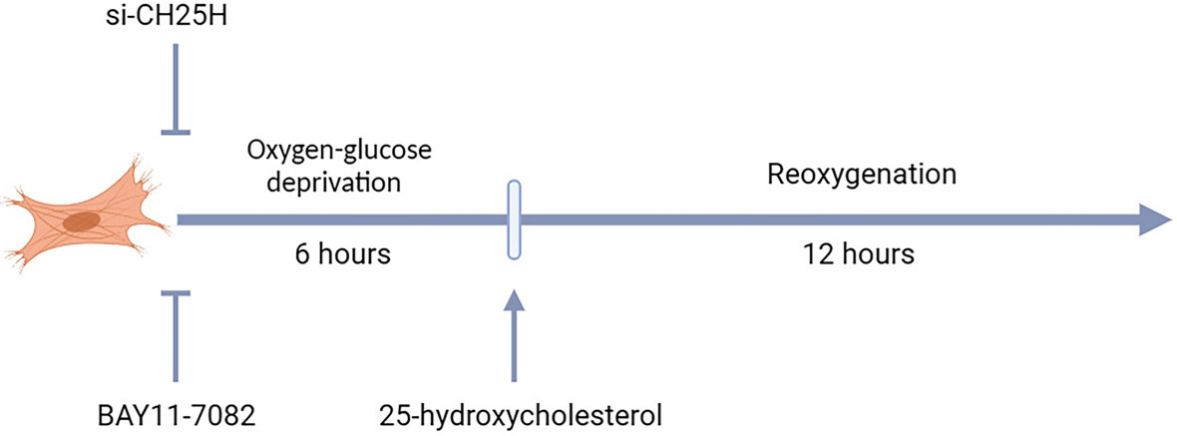
H9C2 cardiomyocytes were subjected to 6 h of oxygen-glucose deprivation and 12 h of reoxygenation (OGD/R). H9C2 cardiomyocytes were transfected with siRNA and then subjected to OGD/R. 25-hydroxycholesterol was used during reoxygenation. The inflammasome inhibitor BAY11-7082 was used 2 h before OGD onset.

The 25-hydroxycholesterol (MCE, USA) was dissolved in dimethyl sulfoxide (DMSO) and used at different concentrations (0.4, 2, 10, or 50 μM) during reoxygenation. The inflammasome inhibitor BAY11-7082 (MCE) was dissolved in DMSO and used 2 h before the OGD onset at a concentration of 2 μM (Figure 1).

### Small interfering RNA transfection

The small interfering RNA (siRNA) that targeted CH25H was purchased from GenePharma (China). H9C2 cardiomyocytes were transfected with the siRNA using Lipofectamine^TM^ 3000 (Invitrogen, USA). When cell confluence reached 70-90%, the diluted Lipofectamine™ 3000 and siRNA were mixed and incubated for 15 to 20 min at room temperature. Then, the complexes were added to the medium for transfection. After incubation for 48 h, the CH25H expression was evaluated using real-time quantitative PCR (RT-qPCR) and western blot assays.

### RT-qPCR analysis

Total RNA was extracted from H9C2 cardiomyocytes using RNA Simple Total RNA kit (Tiangen, China). The complementary DNA (cDNA) was synthesized from 500 ng of total RNA using PrimeScript RT Master Mix (Takara, Japan). Quantitative PCR was performed using SYBR Green qPCR Master Mix (Bimake, China) on Applied Biosystems Quantstudio Dx (Thermo Fisher, USA). The expression of glyceraldehyde-3-phosphate dehydrogenase (GAPDH) was used as an internal reference and the relative expression of target genes was calculated by the 2^-ΔΔCt^ method. The following primer sequences were used: GAPDH forward: 5′ GTGCTGAGTATGTCGTGGAGTC 3′, reverse: 5′ TTGCTGACAATCTTGAGGGA 3′; CH25H forward: 5′ AATACATGAGCGTCTGGGAGC 3′, reverse: 5′ CCACGGAAAGTCGTAACCTG 3′; NLRP3 forward: 5′ TGACGCTCTGTGAGGTTCTG 3′; reverse: 5′ TCAGCTCAGGCTTTTCCTCC 3′; Caspase-1 forward: 5′ CGGGCAAGCCAGATGTTTAT 3′; reverse: 5′ AATGCGCCACCTTCTTTGTT 3′; GSDMD forward: 5′ TCCAGTGCCTCCATGAATGT 3′; reverse: 5′ GTGATCTGCACCTCCTCCTT 3′; IL-1β forward: 5′ AGGCTGACAGACCCCAAAAG 3′; reverse: 5′ CTCCACGGGCAAGACATAGG 3′.

### Western blot analysis

H9C2 cardiomyocytes were lysed in iced RIPA buffer (Solarbio, China) containing protease inhibitors for 30 min and centrifuged at 13523 *g*, 4°C for 10 min to collect the supernatants. The protein concentration was measured by the BCA protein assay kit (Solarbio). Proteins were then separated in sodium dodecyl sulfate (SDS) polyacrylamide gel by electrophoresis and transferred to polyvinylidene fluoride membranes (Millipore, USA). Membranes were blocked with tris-buffered saline with tween 20 (TBST) buffer containing 5% nonfat milk and then incubated with primary antibodies against GAPDH (1:1000, Abcam, UK), Caspase-1 (1:1000, Abcam), NLRP3 (1:1000, Proteintech, China), GSDMD (1:1000, Biorbyt, UK), IL-1β (1:1000, Beyotime, China), and CH25H (1:500, Santa Cruz, USA) overnight at 4°C. After washing with TBST buffer three times, membranes were incubated with secondary antibody (Cell Signaling Technology, USA) for 2 h. The specific protein bands were detected using the Odyssey CLx imaging system (LI-COR, USA).

### Cell viability assay

Cell viability was measured in 96-well plates using the cell counting kit-8 (CCK-8) (Beyotime, China) according to the manufacturer's protocol. A total of 10 μL of CCK-8 reagent was added to each well, followed by incubation for 2 h at 37°C in the dark. The absorbance values were determined at 450 nm with the Synergy H1 microplate reader (Gene Company Limited, China).

### Cytotoxicity assay

Lactate dehydrogenase (LDH) in the supernatants was measured by an LDH cytotoxicity detection kit (Beyotime) to evaluate the cell injury according to the manufacturer's instructions. The absorbance values were determined at 490 nm with the microplate reader.

### Hoechst 33342/propidium iodide fluorescent staining

H9C2 cardiomyocytes were stained with Hoechst 33342 and propidium iodide (PI) in 6-well plates to assess pyroptosis. After different stimulations, cells were stained with 5 μL Hoechst and 5 μL PI (Solarbio) per well for 30 min at 4°C in the dark. The images were obtained immediately and analyzed using a Ti2-U fluorescence microscope (Nikon, Japan). The nuclei were stained blue by Hoechst 33342, and the pyroptosis cells were stained red by PI.

### Statistical analysis

The results were analyzed using one-way ANOVA with Tukey multiple comparison test in GraphPad Prism version 5.0 (USA). Data are reported as means±SD. P values <0.05 were considered to be statistically significant.

## Results

### Identification of DEGs and GO enrichment analysis

**Figure 2:**
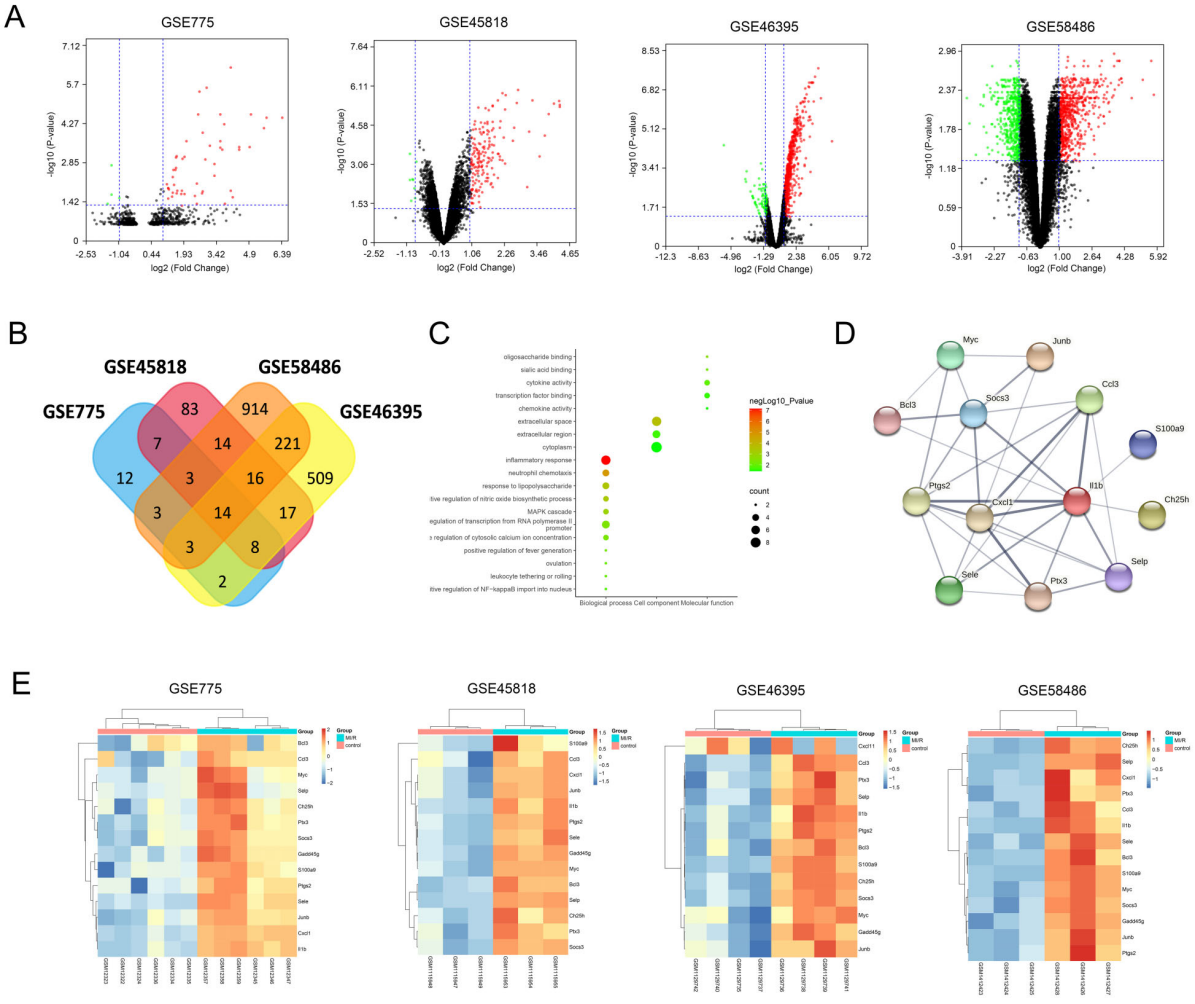
Bioinformatics analysis of Gene Expression Omnibus (GEO) datasets. **A**, Volcano plot of genes from GSE775, GSE45818, GSE46395, and GSE58486. The downregulated genes are represented by green dots (P<0.05 and log (fold change) ≤1), while the upregulated genes were indicated by red dots (P<0.05 and log (fold change) >1). **B**, Venn plot of differentially expressed genes from GSE775, GSE45818, GSE46395, and GSE58486. **C**, Gene ontology enrichment analysis of 14 overlapping differentially expressed genes was conducted using the DAVID database, including terms of biological process, cellular component, and molecular function. **D**, The protein-protein interaction network of 14 overlapping differentially expressed genes was established using the STRING database. **E**, The hierarchical cluster analysis of 14 overlapping differentially expressed genes in GSE775, GSE45818, GSE46395, and GSE58486.

A total of 52 DEGs were identified from the GSE775 dataset, 162 DEGs were acquired from the GSE45818 dataset, 1188 DEGs were identified from the GSE58486 dataset, and 790 DEGs were acquired from the GSE46395 dataset (Figure 2A). After comparing the DEGs of four datasets, 14 overlapping DEGs were identified, including CH25H, IL-1b, and CXCL1 (Figure 2B). The GO and KEGG enrichment analyses indicated that the 14 overlapping genes were mostly related to inflammation, such as inflammatory response (FDR: 7.00E-08), neutrophil chemotaxis (FDR: 1.50E-05), and cytokine activity (FDR: 1.10E-02) (Figure 2C). For these overlapping genes, the PPI network was constructed using the STRING database (Figure 2D) and the hierarchical cluster analysis was performed based on the gene expression (Figure 2E).

### OGD/R promoted CH25H expression and pyroptosis

**Figure 3:**
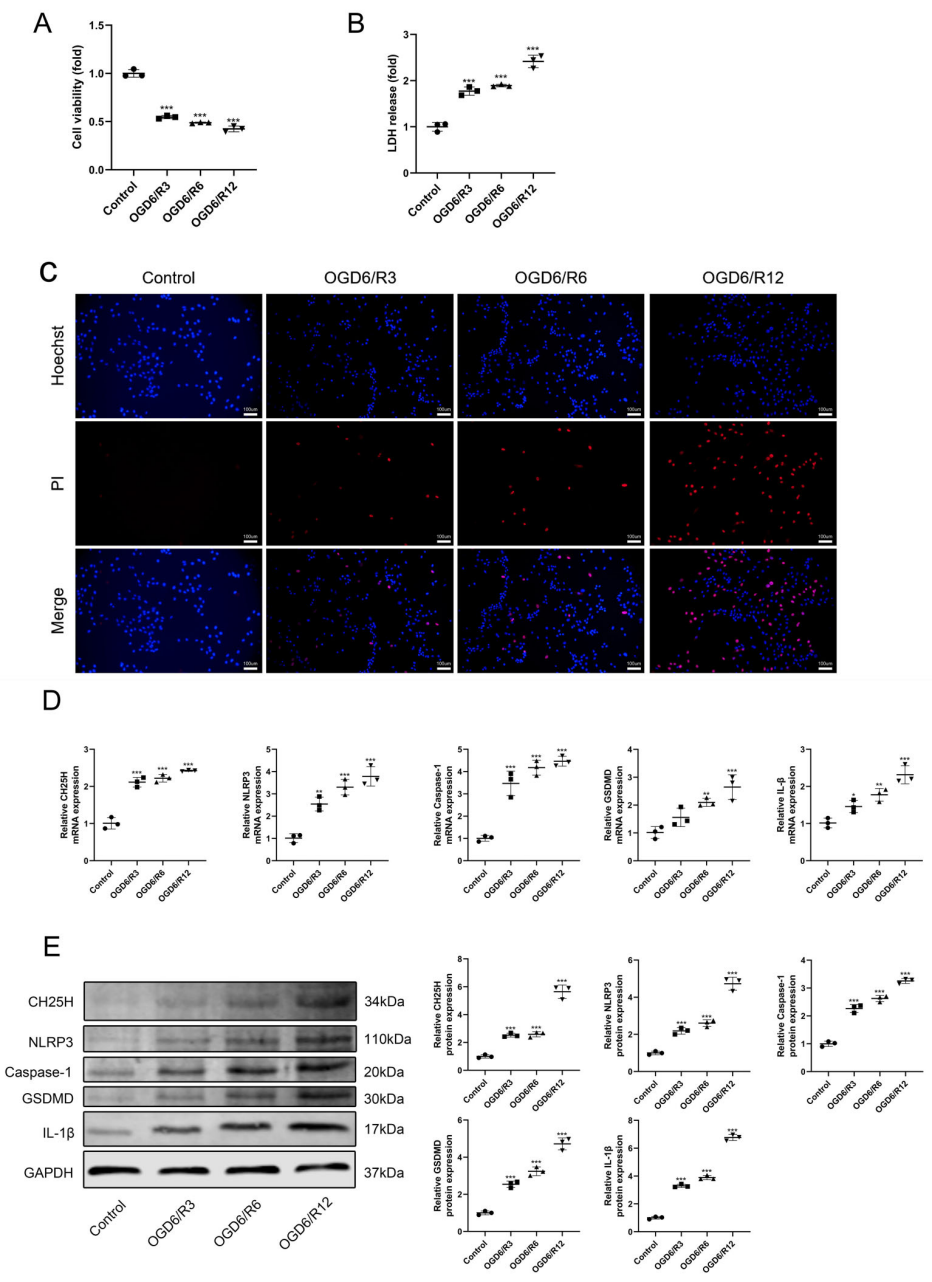
Oxygen-glucose deprivation/reoxygenation (OGD/R) treatment promoted pyroptosis, NLRP3 inflammasome activation, and 25-hydroxylase (CH25H) expression in H9C2 cells. The H9C2 cells were subjected to oxygen-glucose deprivation for 6 h and then subjected to normoxic conditions for 3, 6, or 12 h. **A**, CCK-8 assay was used to detect cell viability. **B**, Lactate dehydrogenase (LDH) cytotoxicity detection assay was used to detect the LDH release. **C**, Hoechst 33342 and propidium iodide (PI) were used to stain cells. The nuclei were stained blue by Hoechst 33342, and the pyroptotic cells were stained red by PI; scale bars=100 μm. **D**, The mRNA and (**E**) protein expressions of CH25H, NLRP3, Caspase-1, GSDMD, IL-1β, and GAPDH were detected by RT-qPCR and western blotting. Data are reported as means±SD (n=3). *P<0.05, **P<0.01, and ***P<0.001 *vs* the control group (one-way ANOVA with Tukey multiple comparison test).

CH25H was highly expressed in these overlapping genes, but its role in MI/R injury remains unclear. To explore the effect of OGD/R treatment on CH25H expression and pyroptosis, the cell viability, LDH release, and expression of CH25H, NLRP3, caspase-1, GSDMD and IL-1β were detected. The OGD6/R12 group had the lowest cell viability (Figure 3A) but the highest LDH release (Figure 3B). The Hoechst33342/PI staining showed that the OGD6/R12 group had more pyroptosis cells than other groups (Figure 3C). Compared with the control group, the mRNA and protein expressions of CH25H were significantly increased after OGD/R exposure (Figure 3D and E), and the CH25H expression gradually increased with reoxygenation time. Importantly, RT-qPCR and western blotting demonstrated that mRNA and protein expressions of NLRP3, caspase-1, GSDMD, and IL-1β were higher in cells of the OGD6/R12 group than in the other groups (Figure 3D and E). Therefore, OGD6/R12-treated H9C2 cardiomyocytes were used for subsequent experiments.

### Downregulation of CH25H expression inhibited the OGD/R-induced pyroptosis and NLRP3 inflammasome activation

**Figure 4:**
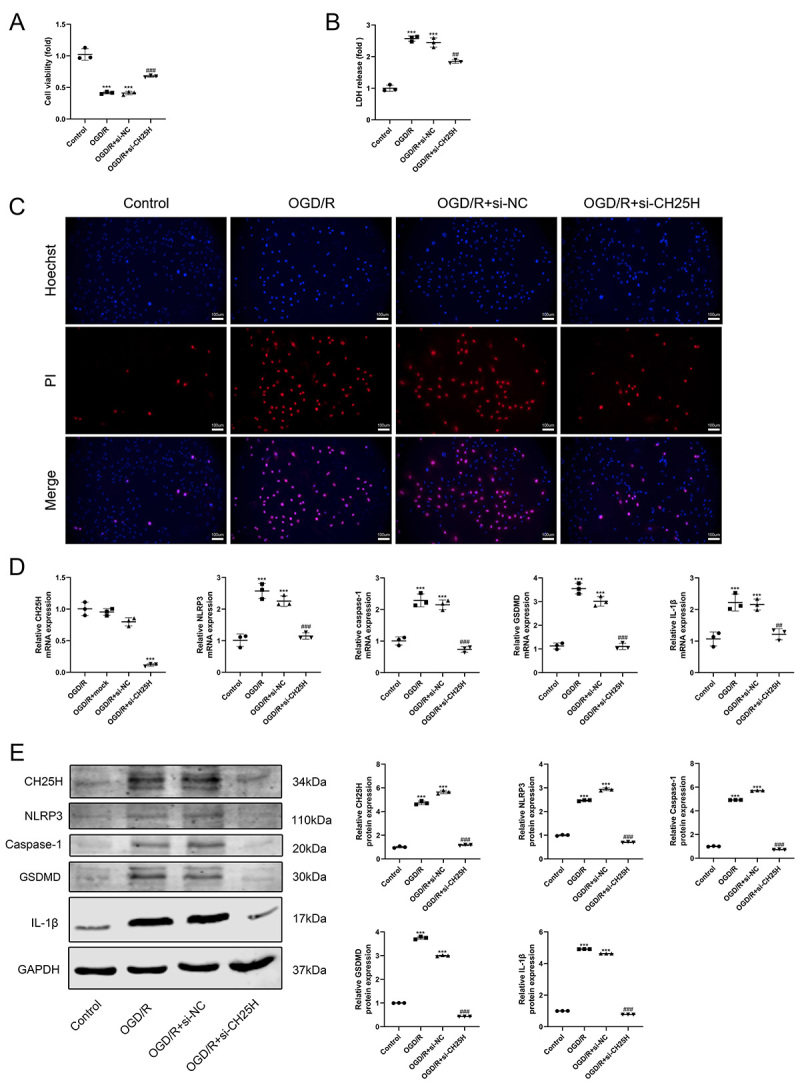
CH25H knockdown inhibited pyroptosis and NLRP3 inflammasome activation in oxygen-glucose deprivation/reoxygenation (OGD/R)-treated H9C2 cardiomyocytes. The H9C2 cells were transfected with si-NC (negative control) or si-CH25H and then subjected to oxygen-glucose deprivation for 6 h following reoxygenation for 12 h. **A**, CCK-8 assay was used to detect cell viability. **B**, Lactate dehydrogenase (LDH) cytotoxicity detection assay was used to detect LDH release. **C**, Hoechst 33342 and propidium iodide (PI) were used to stain cells; scale bars=100 μm. The nuclei were stained blue by Hoechst 33342, and the pyroptotic cells were stained red by PI. **D**, The mRNA and (**E**) protein expressions of CH25H, NLRP3, Caspase-1, GSDMD, IL-1β, and GAPDH were detected by RT-qPCR and western blotting. Data are reported as means±SD (n=3). ***P<0.001 *vs* control group, ^##^P<0.01 and ^###^P<0.001 *vs* OGD/R+si-NC group (one-way ANOVA with Tukey multiple comparison test).

To investigate the effect of CH25H on OGD/R-induced cell injury, CH25H siRNA was transfected into H9C2 cells to knockdown CH25H expression. Downregulation of CH25H reversed the OGD/R-induced reduction in cell survival (Figure 4A), and CH25H knockdown suppressed the increase of LDH release from OGD/R-stimulated cells (Figure 4B). RT-qPCR and western blotting showed that the mRNA and protein expression of CH25H were significantly downregulated in CH25H siRNA-transfected cells following OGD/R treatment (Figure 4D and E). Moreover, knockdown of CH25 restrained OGD/R-mediated pyroptosis (Figure 4C) and increase of NLRP3, caspase-1, GSDMD, and IL-1β (Figure 4D and E).

### 25-HC promoted OGD/R-induced injury and NLRP3 inflammasome activation

**Figure 5:**
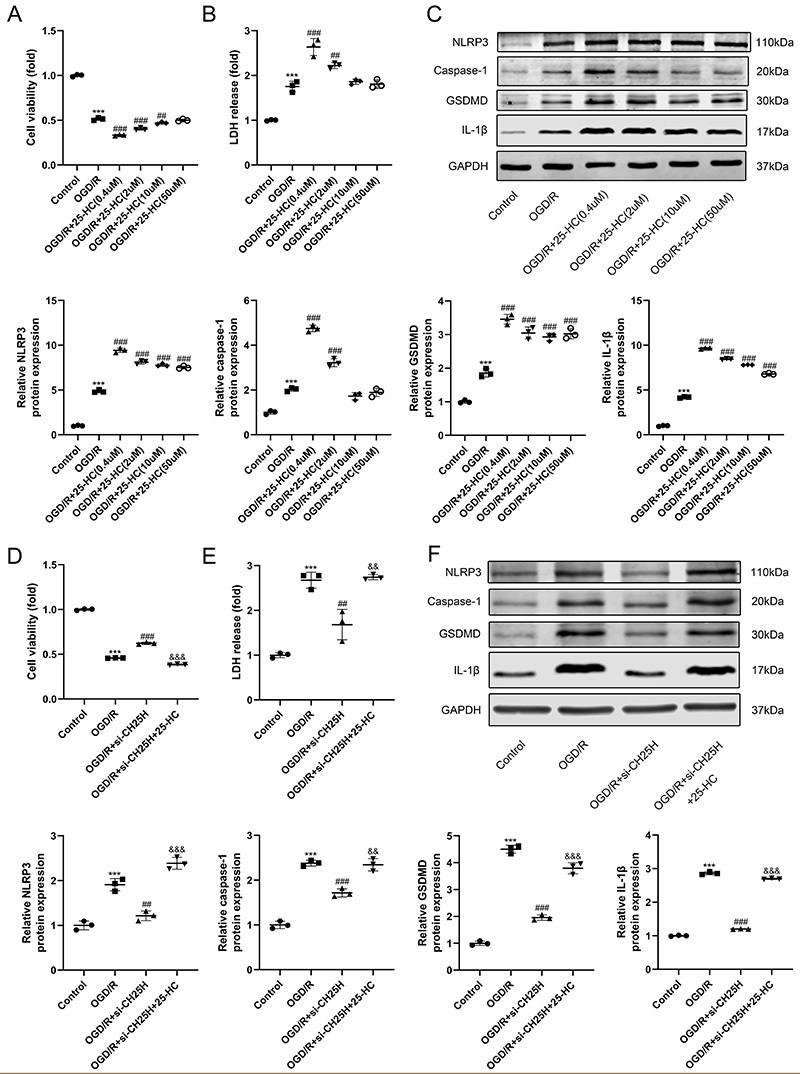
25-HC promoted NLRP3 inflammasome activation in oxygen-glucose deprivation/reoxygenation (OGD/R)-stimulated H9C2 cardiomyocytes. The H9C2 cells were treated with 25-HC at different concentrations (0.4, 2, 10, or 50 μM) during reoxygenation. The si-CH25H-transfected H9C2 cells were treated with 25-HC (0.4 μM) during reoxygenation. **A** and **D**, Cell viability was detected by CCK-8 assay. **B** and **E**, Lactate dehydrogenase (LDH) release was detected by LDH cytotoxicity detection assay. **C** and **F**, The protein expressions of NLRP3, Caspase-1, GSDMD, IL-1β, and GAPDH were detected by western blotting. Data are reported as means±SD (n=3). ***P<0.001 *vs* control group, ^#^P<0.05, ^##^P<0.01, and ^###^P<0.001 *vs* OGD/R group, ^&&^P<0.01 and ^&&&^P<0.001 *vs* OGD/R+si-CH25H group (one-way ANOVA with Tukey multiple comparison test).

25-HC is the synthetic product of CH25H and plays an important role in immunity. To determine the effect of 25-HC on cell injury and NLRP3 inflammasome activation triggered by OGD/R treatment, the cell viability, LDH release, and expression of NLRP3, caspase-1, GSDMD, and IL-1β were detected. 25-HC promoted the decrease of cell viability (Figure 5A) and increase of LDH release induced by OGD/R (Figure 5B). 25-HC also increased the expression of NLRP3, caspase-1, GSDMD and IL-1β in OGD/R-treated H9C2 cells (Figure 5C). Most importantly, 25-HC rescued the effect caused by downregulation of CH25H in OGD/R-treated H9C2 cells. The group treated with CH25H siRNA and 25-hydroxycholesterol had lower cell viability, more LDH release, and higher protein expression of NLRP3, caspase-1, GSDMD, and IL-1β than the group transfected with CH25H siRNA (Figure 5D-F).

### 25-HC promoted OGD/R-induced pyroptosis through activating NLRP3 inflammasome

**Figure 6:**
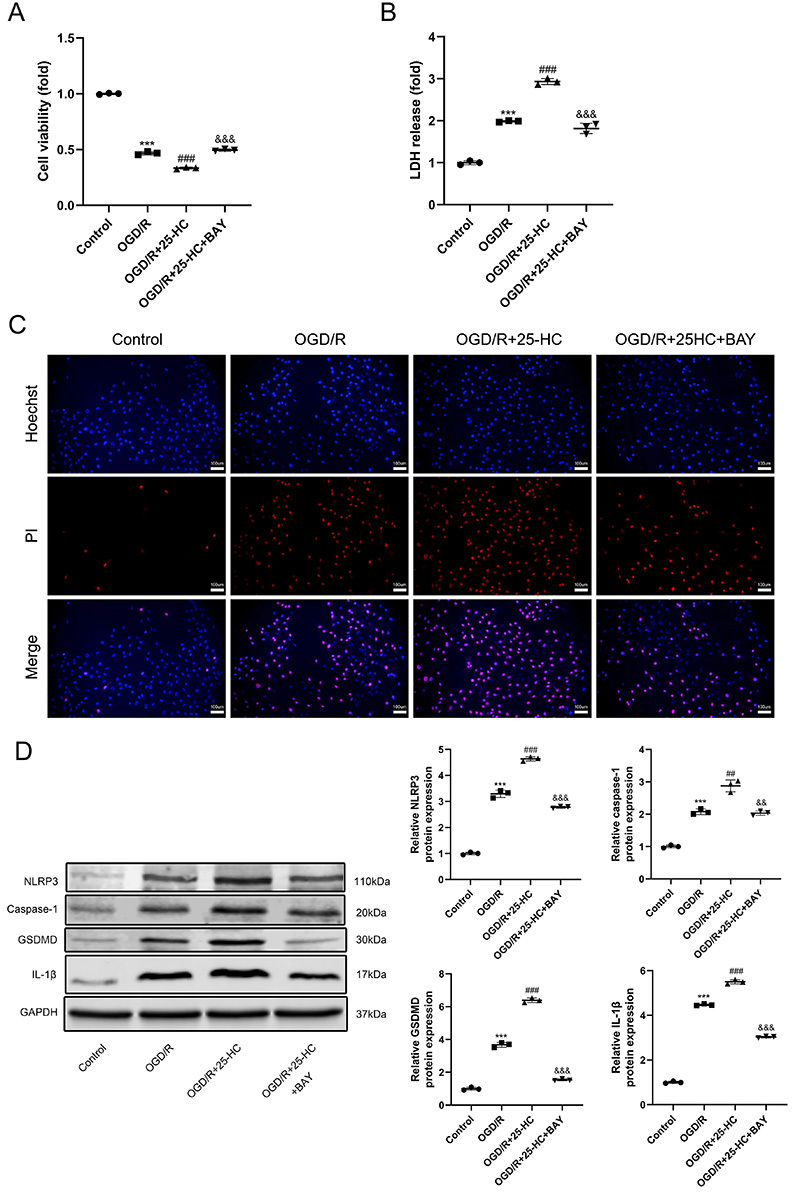
25-HC promoted oxygen-glucose deprivation/reoxygenation (OGD/R)-induced pyroptosis through activation of NLRP3 inflammasome in H9C2 cardiomyocytes. The H9C2cells were pretreated with NLRP3 inflammasome inhibitor BAY11-7082 (2 μM) 2 h before OGD/R treatment. Then, cells were treated with 25-hydroxycholesterol (0.4 μM) during reoxygenation. **A**, CCK-8 assay was used to detect cell viability. **B**, Lactate dehydrogenase (LDH) cytotoxicity detection assay was used to detect the LDH release. **C**, Hoechst 33342 and propidium iodide (PI) were used to stain cells; scale bars=100 μm. The nuclei were stained blue by Hoechst 33342, and the pyroptotic cells were stained red by PI. **D**, The protein expressions of NLRP3, Caspase-1, GSDMD, IL-1β, and GAPDH were detected by western blotting. Data are reported as means±SD (n=3). ***P<0.001 *vs* control group, ^##^P<0.01 and ^###^P<0.001 *vs* OGD/R group, ^&&^P<0.01 and ^&&&^P<0.001 *vs* OGD/R+25-HC group (one-way ANOVA with Tukey multiple comparison test).

To investigate the role of NLRP3 inflammasome in 25-HC-aggravated pyroptosis after OGD/R treatment, BAY11-7082 was used to inhibit NLRP3 inflammasome activation. Treatment of OGD/R-stimulated H9C2 cells with BAY11-7082 attenuated the enhancing effect of 25-HC on the decrease of cell viability (Figure 6A). Also, BAY11-7082 inhibited the release of LDH and pyroptosis in 25-HC-treated H9C2 cells under OGD/R condition (Figure 6B and C). Western blotting showed that the NLRP3 inflammasome inhibitor eliminated the positive effect of 25-HC on the NLRP3, caspase-1, GSDMD, and IL-1β expression in OGD/R-treated H9C2 cells (Figure 6D).

## Discussion

**Figure 7:**
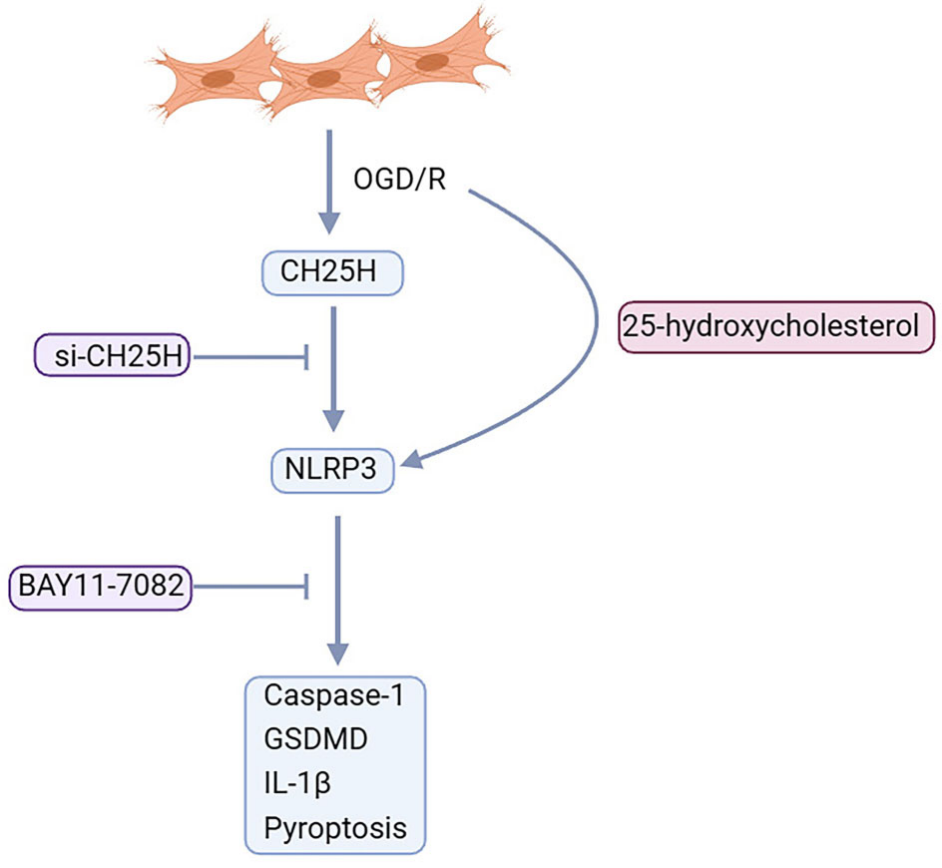
Oxygen-glucose deprivation/reoxygenation (OGD/R) induced CH25H expression, NLRP3 inflammasome activation, and pyroptosis in H9C2 cardiomyocytes. Transfection with CH25H siRNA mitigated OGD/R-induced pyroptosis and activation of NLRP3 inflammasome. However, 25-HC abolished the protective effect of CH25H siRNA. The inflammasome inhibitor BAY11-7082 effectively alleviated OGD/R-induced pyroptosis and NLRP3 inflammasome activation.

In the present study, an OGD/R model was used to simulate the cellular environment during MI/R, which could efficiently induce CH25H expression, NLRP3 inflammasome activation, and pyroptosis in H9C2 cardiomyocytes. Transfection with CH25H siRNA mitigated OGD/R-induced pyroptosis and activation of NLRP3 inflammasome. However, 25-HC abolished the protective effect of CH25H siRNA. The inflammasome inhibitor BAY11-7082 effectively alleviated OGD/R-induced pyroptosis and NLRP3 inflammasome activation. These observations suggested that 25-HC might aggravate OGD/R-induced pyroptosis through promoting activation of NLRP3 inflammasome in H9C2 cardiomyocytes (Figure 7).

25-HC plays a role in the regulation of cell survival and immunity. Previous studies have shown that 25-HC induced a decrease in B-cell lymphoma-2 (BCL-2) expression, an increase in BCL2-associated X (BAX) expression, and an activation of caspases (8, 9, and 3) (17). In addition, 25-HC impaired endothelial function via inhibiting endothelial nitric oxide synthase (eNOS) activity and mediating endothelial cell apoptosis (18). Furthermore, 25-HC promoted cardiomyocyte apoptosis through the caspase-3-dependent mechanism involving activation of the pro-apoptotic protein Bax and degradation of the anti-apoptotic protein Bcl-2 (19). In our study, 25-HC decreased the viability of OGD/R-treated cardiomyocytes by promoting pyroptosis. However, a recent study showed that 25-HC attenuated MI/R injury through suppressing poly ADP-ribose polymerase (PARP) activity and reducing cardiomyocyte apoptosis (20).

Several studies have demonstrated that 25-HC has pro-inflammation and anti-inflammation effects. 25-HC was utilized to maintain mitochondrial integrity and prevent AIM2 inflammasome activation in activated macrophages (10). 25-HC also inhibited IL-1β transcription and IL-1-activated inflammasomes by antagonizing sterol response element-binding protein (SREBP) processing (9). Moreover, in lipopolysaccharide (LPS)-induced acute lung injury, 25-HC suppressed the overwhelming inflammatory response via myeloid differentiation protein 2 interaction, which inhibited the Akt/NF-κB signaling pathway (21).

Contrary to previously described findings, several studies have shown that 25-HC contributed to the release of inflammatory cytokines and activation of inflammasomes. Some studies reported that 25-HC increases the production of proinflammatory cytokines such as IL-8, IL-6, monocyte chemotactic protein (MCP)-1, and tumor necrosis factor (TNF)-α (11,22-25). Moreover, CH25H expression was upregulated, and 25-HC promoted IL-1β-mediated neuroinflammation in brain tissue of patients with Alzheimer's disease (26). CH25H and 25-HC production also increased in childhood cerebral adrenoleukodystrophy, and 25-HC promoted IL-1β production in an NLRP3 inflammasome-dependent manner (12). Furthermore, CH25H expression was significantly increased in lung tissue of chronic obstructive pulmonary disease (COPD) patients. The concentration of 25-HC in the sputum of COPD patients was significantly elevated and significantly correlated with sputum IL-8 levels and neutrophil counts (27). Consistent with the above studies, our findings showed that CH25H expression was upregulated in OGD/R-treated H9C2 cardiomyocytes and that 25-HC promoted NLRP3 inflammasome activation and IL-1β production.

However, there were several limitations in this study. This study did not show the morphological changes of pyroptotic cells and further research should assess cell morphology after different treatments. A relatively small sample size was used in this study. Pro-inflammatory cytokines other than IL-1β were not investigated in this study. To investigate the effect of 25-HC on inflammation in MI/R injury, more pro-inflammatory cytokines such as IL-6 and IL-18 should be evaluated in further studies. This study only explored the role of BAY11-7082, an inhibitor of the NLRP3 inflammasome, to investigate the effects of NLRP3 inflammasome activation in MI/R injury. Thus, further studies are needed to investigate the effects of other specific inflammatory inhibitors in MI/R injury to verify the specific effects of NLRP3 inflammasome.

In conclusion, our findings demonstrated that 25-HC decreased cell viability and promoted pyroptosis through activating NLRP3 inflammasome in OGD/R-stimulated cardiomyocytes. Our study suggested that 25-HC could aggravate cell damage and might play an important role in MI/R injury.
